# Functional MRI Representational Similarity Analysis Reveals a Dissociation between Discriminative and Relative Location Information in the Human Visual System

**DOI:** 10.3389/fnint.2016.00016

**Published:** 2016-03-30

**Authors:** Zvi N. Roth

**Affiliations:** ^1^The Edmond and Lily Safra Center for Brain Sciences, The Hebrew UniversityJerusalem, Israel; ^2^Department of Neurobiology, The Hebrew UniversityJerusalem, Israel

**Keywords:** representational similarity analysis (RSA), multivariate pattern analysis (MVPA), relative information, vision, functional magnetic resonance imaging (fMRI)

## Abstract

Neural responses in visual cortex are governed by a topographic mapping from retinal locations to cortical responses. Moreover, at the voxel population level early visual cortex (EVC) activity enables accurate decoding of stimuli locations. However, in many cases information enabling one to *discriminate* between locations (i.e., discriminative information) may be less relevant than information regarding the *relative* location of two objects (i.e., relative information). For example, when planning to grab a cup, determining whether the cup is located at the same retinal location as the hand is hardly relevant, whereas the location of the cup relative to the hand is crucial for performing the action. We have previously used multivariate pattern analysis techniques to measure discriminative location information, and found the highest levels in EVC, in line with other studies. Here we show, using representational similarity analysis, that availability of discriminative information in fMRI activation patterns does not entail availability of relative information. Specifically, we find that relative location information can be reliably extracted from activity patterns in posterior intraparietal sulcus (pIPS), but not from EVC, where we find the spatial representation to be warped. We further show that this variability in relative information levels between regions can be explained by a computational model based on an array of receptive fields. Moreover, when the model's receptive fields are extended to include inhibitory surround regions, the model can account for the spatial warping in EVC. These results demonstrate how size and shape properties of receptive fields in human visual cortex contribute to the transformation of discriminative spatial representations into relative spatial representations along the visual stream.

## Introduction

Among the many features belonging to visual stimuli, location has often been viewed as unique and different from other stimulus properties such as color or shape. For example, it is commonly suggested that the location of an object is the property that enables the binding of all other properties of that object into a single percept (Treisman and Gelade, 1980; Golomb et al., 2014). Other studies suggest that location guides feature binding in working memory, as well as in perception (Treisman and Zhang, 2006; Pertzov and Husain, 2014). Clearly, information regarding the locations of visual stimuli is present already in the retina—retinal ganglion cells respond to stimuli in their receptive fields, and therefore knowing which cells are firing can inform us about stimuli locations (Schwartz et al., 2012; Marre et al., 2014). Location information is abundant in visual cortex as well, as is evident from retinotopic mapping (Wandell et al., 2007).

Many studies have distinguished between retinal and extra-retinal (e.g., head-centered, hand-centered, or allocentric) coordinate frames. While early visual cortex (EVC) seems to make use solely of retinal coordinates, higher order areas may encode stimuli in other coordinate frames that are more relevant to those regions' functions (Andersen et al., 1997; Colby and Goldberg, 1999; Cohen and Andersen, 2002). For example, neurons in LIP, involved in eye movements, represent the world in head-centered coordinates (Andersen et al., 1985).

One way such extra-retinal coordinate systems can come into being, is by representing space in a way that preserves the location of each object (e.g., a cup) relative to other objects (e.g., the arm, in the case of arm-centered coordinates). To guide us in testing whether this is indeed the case in visual cortex, we suggest here a different distinction, between discriminative and relative location information. Discriminative information enables one (e.g., a human observer presented with stimuli or a decoder supplied with brain activity vectors) to determine whether two stimuli were presented in the same location (in any given coordinate system), whereas relative information is used when determining the location of one stimulus relative to another.

Discriminative location information has been studied extensively. Discriminative information can be measured by testing the ability of decoders to discriminate between different locations based on fMRI activation patterns (Kravitz et al., 2010; Golomb and Kanwisher, 2012; Cichy et al., 2013; Roth and Zohary, 2015b). However, since spatial relations between objects are crucial for performing object-related actions, relative information is, in many cases, far more relevant than discriminative information when performing everyday tasks. Nevertheless, relative information has received less attention than its discriminative counterpart (for exceptions see: Hayworth et al., 2011; Sereno and Lehky, 2011; Baeck et al., 2013).

It may initially seem that there is really no fundamental difference between discriminative and relative information, since given the accurate locations of two objects (i.e., discriminative information) one can easily calculate the distance between them by simple vector subtraction. However, this is not always the case. To stress this point, consider a hypothetical example where every point in space is numbered randomly. Given objects at two locations (say, location #3 and location #21) it is easy to determine whether or not they reside in the same location or not (in the above example they do not). However, this accurate discriminative information may not provide any clue as to the distance between the objects (without additional information regarding distances between the locations). A less trivial scenario that will further illustrate this point will be presented later on, when we analyze location information in EVC.

Beyond a technical definition that distinguishes discriminative from relative information, is there any difference between the two types of information at a neural level? Here, we set out to compare the physiological basis of these two types of information. In a previous study we measured the extent of *discriminative* location information in several cortical regions, using fMRI multivoxel pattern analysis (MVPA) methods (Roth and Zohary, 2015b). Here, we use representational similarity analysis (RSA), another MVPA method (Kriegeskorte et al., 2008a; Kriegeskorte and Kievit, 2013), to uncover *relative* location information across those cortical regions, and compare the results with those obtained for discriminative information. We further extend our analysis to quantify both the relative distance and relative *direction* between objects (i.e., relative displacements). Our results show that although EVC was found previously to carry the highest levels of discriminative information, it shows low levels of relative information, with a distorted spatial layout of distances and displacements. These findings suggest a functional dissociation between the two types of location information. Finally, we present a simple computational model to explain the cause of the observed spatial warping in EVC.

## Materials and methods

### Subjects

Fifteen healthy right-handed subjects (4 females) gave their informed consent to participate in the fMRI study, which was approved by the Helsinki Ethics Committee of Hadassah Hospital, Jerusalem, Israel. One subject (male) was excluded from further analysis due to excessive head movement during one of the scans. Thus, data from 14 subjects was used in this study.

### Stimuli

Each of six 1800 ms video clips depicting a right hand grasping (i.e., taking hold) and using a tool (hammer, screwdriver, stapler, corkscrew, garlic press, or knife), was downscaled to 140 × 140 pixels [2.18 × 2.18 degrees of visual angle, or 1 × 1 stimulus widths (SWs)]. The original clips featured a right hand, and by creating mirror-image clips six left-hand clips were generated, resulting in a total of 12 different clips.

### Experimental design and tasks

**Figure 1:**
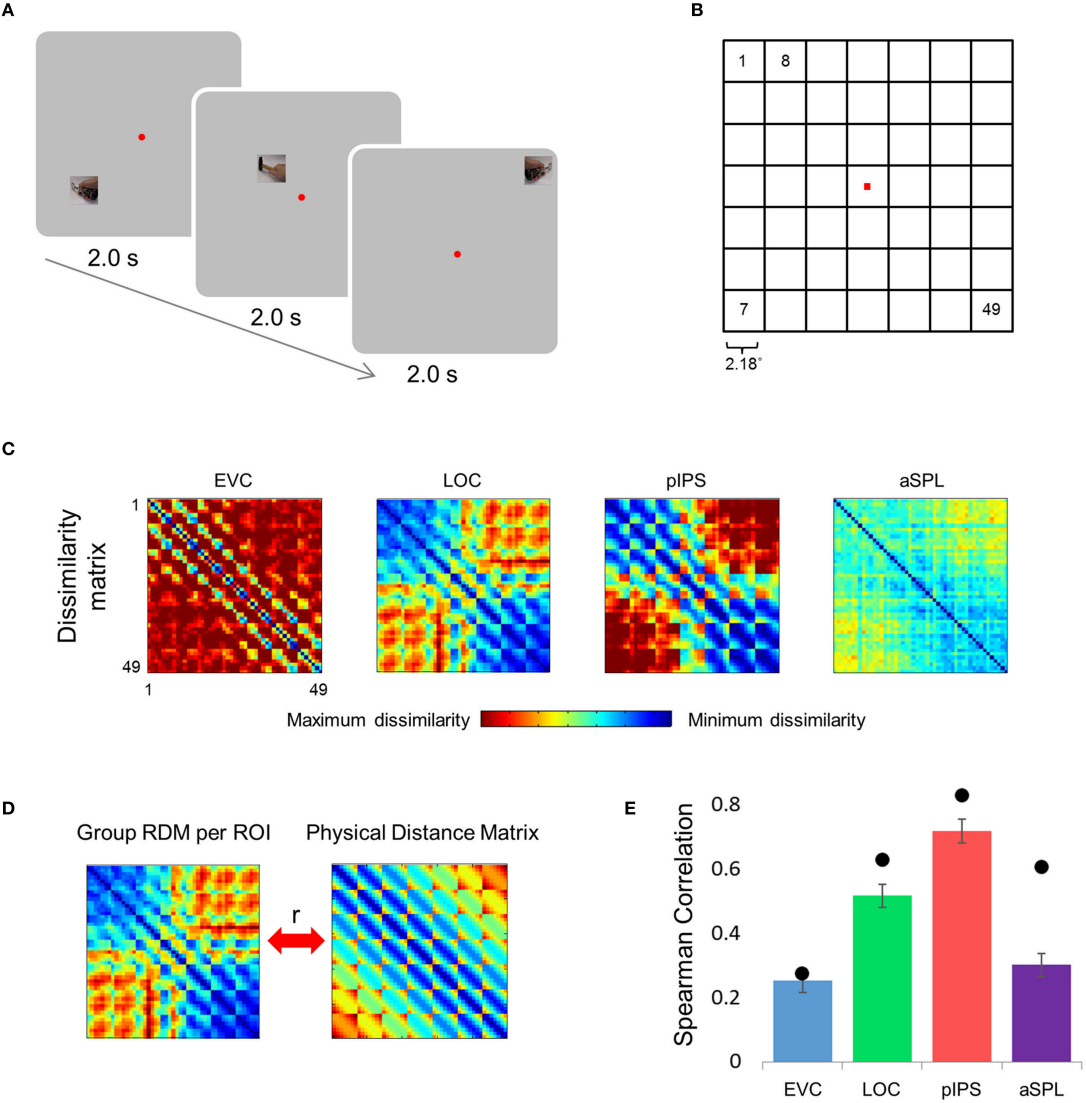
**Relative location information analyzed by representational similarity analysis. (A)** Experimental Design. While fixating a central point, subjects were presented with short video clips depicting a hand grabbing and manipulating a tool—stimuli that activate both ventral and dorsal visual streams—and were instructed to covertly name both the hand (left or right) and the tool. **(B)** Numbering of the 49 locations in the order in which they appear in the correlation matrices. Note that the exact same clips were shown in each of the locations. **(C)** Group Representational Dissimilarity Matrices (RDMs) for each ROI, averaged across all subjects' individual matrices. Each location is represented by a vector of activation levels across voxels within a specific ROI. The RDM contains dissimilarity values (one minus the correlation) between all pairs of location representations. Note that in EVC, pIPS, and aSPL the values are smallest along the main diagonal (same position; the dissimilarity equals 0), and are relatively small also along the parallel lines that reflect the nearest neighbors in terms of stimulus location. **(D)** We measure the informativeness of an ROI by correlating (Spearman's r) the RDM with the physical distance matrix (PDM): the higher the correlation, the better the correspondence between neural dissimilarities and physical distances. **(E)** Spearman correlation results. Bar values and error-bar values, here and in subsequent figures, represent the correlations obtained for single subject RDM results, mean across subjects ± the standard error of the mean. Black points, here and in subsequent figures, represent the group RDM results.

Each subject completed eight runs of the main experiment and a localizer run. During the main experiment subjects fixated on a central red square while viewing video clips at various locations on the screen (Figure 1A). Adjacent stimulus locations were spaced 1 SW (2.18°) from each other, so that the different stimulated locations did not overlap (Figure 1B). Subjects were instructed to covertly name both the hand (left or right) and the tool in each clip. During each trial a single clip was presented, enabling subjects to direct their full attention to that clip (see Section Discussion). The sequential presentation also made it easier to separate the responses to the different stimuli. Each session began with a training period during which subjects at first were allowed to gaze directly at each presented clip, until they felt acquainted with them, and they then practiced keeping central fixation while paying attention to the clips and covertly naming them. Once the subjects reported feeling comfortable with the task, the scanning commenced. Across all eight runs each clip was presented 98 times, twice in each location, and each location hosted 24 clip presentations, three presentations per run. Each trial lasted 2000 ms (1800 ms clip duration with 200 ms ISI), i.e., 1 TR. In addition to 147 clip trials, each run included 49 randomly interspersed null trials during which no clip was presented. Four additional null trials at the beginning and at the end of the run brought the total run duration to 6:48 min (204 volumes). For all analyses presented here we ignored the stimulus identity, focusing instead solely on the stimulus location. Note that while the clip location was variable, the clips remained identical across locations. Thus, we manipulated the location of the clips, not of the location of the tools relative to the hand, which remained constant.

The localizer scan was comprised of 6 blocks of hand, face, animal, tool, and phase-scrambled images. Each block consisted of 32 images, presented for 450 ms each with 50 ms ISI. In each block 0–2 images were repeated consecutively, and subjects indicated such repetitions by button press (i.e., a one-back task). Four initial null trials and four final null trials brought the run duration to a total of 8:16 min (248 volumes). During most of the runs eye movements were recorded and monitored online via a video-based, infra-red eye tracker (Eye Link1000, SR Research, Ontario, Canada).

### MRI scanning parameters

The blood oxygenation level dependent (BOLD) fMRI measurements were obtained using a 3-T Magnetom Skyra Siemens scanner and a 32-channel head coil, at the ELSC Neuroimaging Unit (ENU). The functional MRI protocols were based on a multislice gradient echo-planar imaging and obtained under the following parameters: TR = 2 s, TE = 30 ms, flip angle = 90°, imaging matrix = 64 × 64, field-of-view = 192 mm; 37 slices with 3 mm slice thickness and 15% gap (0.45 mm), were oriented in an oblique position, covering the whole brain, with functional voxels of 3 × 3 × 3 mm. In addition, high-resolution T1-weighted magnetization-prepared rapid acquisition gradient-echo (MPRAGE) images were acquired (1 × 1 × 1 mm resolution). All scans used GRAPPA parallel imaging (acceleration factor = 2).

### Data processing

Data analysis was conducted using the BrainVoyager QX software package (Brain Innovation) and in-house analysis tools developed in Matlab (MathWorks). Preprocessing of functional scans included 3D motion correction, slice scan time correction, and removal of low frequencies (linear trend removal and high-pass filtering). The anatomical and functional images were transformed to the Talairach coordinate system using trilinear interpolation. The cortical surface was reconstructed from the high-resolution anatomical images using standard procedures implemented by the BrainVoyager software.

Voxel time courses were generated using BrainVoyager and were then analyzed using Matlab custom-made software. Specifically, we first transformed each voxel's time course to obtain a z-score value, by subtracting the mean activation and dividing by the standard deviation of the BOLD response across the whole run. Next, we used a standard General Linear Model (GLM) analysis with a regressor for each of the 49 locations, assuming the standard (two-gamma) hemodynamic response function. This resulted in one activation parameter (beta value) per location, for each voxel. We then transformed the beta values into *t*-values, by subtracting each voxel's mean beta value (across all locations) and dividing by each beta's standard deviation (Roth and Zohary, 2015a).

### Region of interest (ROI) selection

In a separate functional localizer subjects viewed images of hands, tools, and scrambled tools in a block-design run. We defined four ROIs: early visual cortex (EVC), lateral occipital complex (LOC), posterior intraparietal sulcus (pIPS), and anterior superior parietal lobule (aSPL). To obtain two separable ROIs in the parietal cortex for each subject, we used the contrast: hands > scrambled and, starting from FDR < 0.001, raised the threshold separately for each hemisphere until a cluster of active voxels in the anterior portion of the parietal cortex was separated from the posterior IPS. We then raised the threshold further (if necessary) until a cluster of pIPS voxels was separated from occipital areas; this defined pIPS. Next, we contrasted tools > scrambled, and starting from FDR < 0.001, raised the threshold until a ventral-occipital cluster separated from the parietal region, and thus selected LOC. Finally, we utilized the fact that early visual areas are sensitive to local contrast that is enhanced in the scrambled images. Thus, the opposite contrast (scrambled > tools) and the same threshold as for LOC was used to select a cluster of voxels in the posterior occipital cortex, defined as EVC.

Since LOC consistently shows information levels somewhere between EVC and pIPS, we do not discuss it specifically in most of the Results Section. We also do not discuss aSPL, as it shows low levels of both discriminative and relative information. We therefore focus mainly on EVC and pIPS. We do, however, present the main results of all four ROIs, to enable an easy comparison with our previous discriminative results (Roth and Zohary, 2015b).

### Representational dissimilarity matrix (RDM)

For each ROI we had 49 vectors (corresponding to the 49 stimulus locations) of N *t*-values each, where N is the number of voxels in the ROI. The RDM is a matrix containing the pairwise dissimilarities between all 49 vectors, i.e., a total of 49 × 49 = 2401 dissimilarity values. To create an RDM (Kriegeskorte and Kievit, 2013) we correlated all 49 vectors with one another (using Pearson correlation) and subtracted the values from 1. This resulted in a matrix M_49 × 49_ of dissimilarity values—i.e., the higher the value of M_ij_, the lower the correlation between location i and location j. We created an RDM for every subject and ROI. Then, for each functional ROI we averaged all 14 single-subject RDMs to create a single group RDM.

### Physical distance matrix (PDM)

Next, we tested whether the dissimilarity between representations of different locations corresponds to the physical distance between them. To do so, we created a physical distance matrix (PDM) in which the values of the matrix were simply the Euclidean distances between the 49 locations. In other words, if *P* is the PDM, then *P*_*ij*_ is the Euclidean distance between location *i* and location *j*. After transforming both the ROI RDMs (single subject RDMs and group RDMs) and the PDM into vectors (each containing 49 × 49 = 2401 values) we calculated the Spearman correlation between the RDMs and the PDM (Kriegeskorte and Kievit, 2013). The higher the correlation, the better the representation dissimilarity between locations corresponds to the actual physical distance between locations. We estimated the statistical significance of the correlations by permuting the values of each group RDM 10^6^ times and counting the number of permutations which yielded correlation values (with the PDM) higher than the actual data. For all ROIs the actual RDM had a higher correlation with the PDM than any of the permuted RDMs did (i.e., the correlations were significant at *p* < 10^−6^ for all ROIs).

### Dissimilarity-distance (DD) function

If a given ROI represents relative distances in a physically accurate fashion, locations further away must have higher dissimilarities between their representative activity vectors than locations at a smaller distance from each other. In order to directly visualize this aspect of the RDM we plotted dissimilarity as a function of distance between locations, averaging across pairs of locations with equal distances.

### Multidimensional scaling (MDS)

As a means to visualize more easily the correspondence between the neural RDM and the physical locations (and distances), the high dimensional RDMs were transformed into 2-dimensional representations by multidimensional scaling (MDS; Shepard, 1980; Sereno and Lehky, 2011). MDS is an algorithm that converts the 49 locations to points in a smaller dimensional space while preserving as well as possible all the relative distances/dissimilarities; locations that have a large dissimilarity between them will generally be further away from each other in the 2D space than location pairs with small dissimilarities. Specifically, MDS was used to project the 49 locations onto a 2 dimensional (2D) space, by using the “mdscale” function in Matlab with the “strain” criterion.

Next, we directly compared the MDS output with the physical layout, both computationally and visually. The MDS output, consisting of 49 points in 2D space, underwent Procrustes transformation, which uses scaling, translation, rotation, and inversion in order to fit all the points from the MDS output to their physical locations. The sum of squares of the difference between the final, transformed, two-dimensional points (corresponding to the neural representations of the 49 location), and the physical locations quantifies how different the MDS output is from the physical layout, and is termed Procrustes Distance (PD). A low PD means that the 2D projection of the RDM corresponds well to the physical distances. Since the MDS function returned slightly different results during different executions (this occurred for the model results when fitted to the EVC RDM), we repeated the MDS and Procrustes analyses 20 times, and chose the result with the lowest PD. The statistical significance of the PD was determined by permuting the labels of the MDS output 10^6^ times and counting the number of permutations which yielded higher PD values than the actual data. For all ROIs, the original MDS output had a lower PD than any of the relabeled MDS outputs. Thus, the correspondence between the MDS output and the physical layout was significant at *p* < 10^−6^, for all four ROIs.

### Computational model

To help us understand the results, we used a computational model consisting of a grid of receptive field (RF) centers, with adjacent RFs spaced 1 SW apart (Lehky and Sereno, 2011; **Figure 4A**). We used 2 versions of this model. In the first version (Gaussian model) each RF is a 2D Gaussian. In the 2nd version (DoG model) each RF is the sum of a positive Gaussian and a wider (larger σ) negative Gaussian, resulting in a RF with a central excitatory region and a surrounding inhibitory region (**Figure 4C**). Such a RF is also known as center-surround or Difference-of-Gaussians (DoG). The Gaussian model has two parameters that we fit to the data: the size of the RFs at fixation, σ_0_, and the rate at which the RF size increases with eccentricity, *a*. Assuming a RF is centered at (*x*′, *y*′), the distance of the center from fixation is $r=\sqrt{{x{}^{\prime }}^{2}+{y{}^{\prime }}^{2}}$ and its response to a stimulus at location (*x, y*) will be: $T\left(x,y\right)=e^{-\left(\frac{{\left(x-x^{\prime }\right)}^{2}}{2\sigma^{2}}+\frac{{\left(y-y^{\prime }\right)}^{2}}{2\sigma^{2}}\right)}$, where σ = σ_0_ + *a* · *r*. The DoG model has four parameters: the size of the positive Gaussian at fixation, $\sigma_{0}^{+}$; the size of the negative Gaussian at fixation, $\sigma_{0}^{-}$; the rate at which the Gaussian size increases with eccentricity (same rate for both Gaussians), *a*; and the ratio of the negative to positive Gaussian amplitudes, *R*. This RF's response will be: *T*(*x, y*) = *T*^+^(*x, y*)+*R*·*T*^−^(*x, y*), where $T^{+}\left(x,y\right)=e^{-\left(\frac{{\left(x-x^{\prime }\right)}^{2}}{2{\sigma^{+}}^{2}}+\frac{{\left(y-y^{\prime }\right)}^{2}}{2{\sigma^{+}}^{2}}\right)}$, $T^{-}\left(x,y\right)={-e}^{-\left(\frac{{\left(x-x^{\prime }\right)}^{2}}{2{\sigma^{-}}^{2}}+\frac{{\left(y-y^{\prime }\right)}^{2}}{2{\sigma^{-}}^{2}}\right)}$, $\sigma^{+}=\sigma_{0}^{+}+a\cdot r$ and $\sigma^{-}=\sigma_{0}^{-}+a\cdot r$.

The number of RFs per array point decreased exponentially with eccentricity (**Figure 4B**). For the simulations presented here, the number of RFs centered at each location, *P*(*r*), was determined by the equation: *P*(*r*) = 40·*e*^−0.3· *r*^, where *r* is the distance from fixation in SWs.

The model was fitted to each RDM, with the goal of maximizing the correlation between the data RDM and the model RDM. Optimal parameters for the model were found with a 2-stage fitting procedure. The first stage, a grid search, involved running the model with a predefined wide range of parameters, and choosing the set of parameters yielding the best results (i.e., highest correlation). At the second stage we performed unconstrained linear optimization, with the Matlab function “fminsearch,” using the parameters found during the first stage as the initial parameters. While the second stage can generally find a local minimum, the grid search performed in the first stage increases the probability that this will also be a global minimum.

### Searchlight analysis

Since our analyses were performed on ROIs we could not be sure that we did not miss any relevant cortical regions. Therefore, as a complementary analysis we applied a searchlight analysis, performing the analysis on cubes of voxels, instead of the pre-defined ROIs (Kriegeskorte et al., 2006). Specifically, the cubes measured 5 × 5 × 5 and 7 × 7 × 7 functional voxels and we used every possible cube of contiguous voxels across the brain volume. As with the ROI-based correlation analysis, we calculated the RDM for each searchlight cube, and computed the correlation between the RDM and the PDM. We then performed MDS on the RDM and calculated the PD. Thus, for every cube center we had two values (the RDM-PDM correlation and the PD), which we mapped across the brain (**Figures 7C,D**). In addition, we computed the discriminative location information with the correlation method (Roth and Zohary, 2015b). Specifically, for each ROI cube we divided the runs into two equally sized groups, concatenated the *z*-scored time-courses within each group and extracted 49 beta coefficients for the 49 locations with the GLM. Next, we correlated the 49 location vectors in one half with the vectors of the other half resulting in a correlation matrix. We then subtracted the mean correlation between different locations from the mean correlation between identical locations, to estimate the level of discriminative location information (Haxby et al., 2001; Roth and Zohary, 2015a). We repeated this procedure 12 times, each time dividing the runs differently into two groups. The averaged results from all data divisions were taken as a measure of discriminative location information, mapped across the brain (**Figure 7B**).

### Displacement population vectors

Finally, to test the possibility of direction representation we created additional population vectors by way of subtraction (**Figure 8**). For each of the 49 location vectors, we subtracted all other 48 location vectors, resulting in 49 × 48 = 2352 “synthetic” population vectors (MacEvoy and Epstein, 2009) that are the population changes corresponding to location changes. We hypothesized that these vectors may represent the location changes, or displacement vectors. Using these synthetic displacement vectors, we created new displacement-RDMs (dRDMs) with 2352 × 2352 values. In order to create the displacement-PDM (dPDM) we performed subtraction on the corresponding 2D physical vectors, and then aligned the results with the origin, so that for each synthetic vector we had a physical vector representing the physical displacement (both angle and magnitude). We then correlated the dRDM with the dPDM. Next, we performed MDS on the dRDM, and used Procrustes tranformation to fit the MDS result to the physical layout of displacement vectors, quantifying the fit with PD. These analyses were performed at both the single-subject and the group levels, similar to the analyses above performed on location vectors. Once again, the higher the correlation between the dRDM and the dPDM, and the lower the PD between the MDS result and the physical layout of the location displacements, the better the neural representations of these changes correspond to the physical changes.

## Results

Participants were scanned while viewing short video clips presented at 49 different retinal/screen locations (Figures 1A,B). We focused on the similarity between multivoxel patterns (i.e., representations) evoked by stimuli at different locations, and the degree to which such similarity corresponds (inversely) to the physical distance between locations. Aside from the location at which the clips were presented, we did not manipulate any properties of the clips, which remained constant across locations. We studied regions across the visual system, in both the ventral and the dorsal streams: EVC, LOC, and parietal areas pIPS, and aSPL.

### Correlation between the representational dissimilarity matrix (RDM) and the physical distance matrix (PDM)

Can the distance between two locations be inferred from the similarity of their representations? To answer this question we created an RDM for each ROI (Figure 1C). The RDM contains the dissimilarity values (defined here as 1-r, where r is Pearson's correlation) for all pairs of locations. Each row of the RDM contains the dissimilarity values between a certain location (corresponding to the specific row chosen) and all the 49 locations.

Next, we correlated the RDM of each ROI with the PDM, which contains the physical Euclidean distances between all pairs of locations (Figure 1D). If the dissimilarities in a certain ROI are determined by the distances between locations, we should find a close match between the RDM and the PDM. Therefore, a high correlation between the RDM and the PDM would indicate that the location representations preserve relative distances. If discriminative and relative location information depend on one another, EVC should show the highest degree of relative information as it has the highest degree of discriminative information (among the four ROIs tested; see also Searchlight Analysis). However, on the contrary, we found that while the pIPS RDM has the highest correlation to the PDM, EVC shows the lowest correlation (Figure 1E). In other words, although EVC has the highest degree of discriminative information, it has the lowest degree of relative location information.

### Multidimensional scaling (MDS)

To visualize the correspondence between RDMs and the PDM, we used MDS to transform the 49 locations to points in 2D space, such that the distances between points will match the corresponding dissimilarity values as best as possible (i.e., points with larger dissimilarity values reside further away from one another than points with smaller dissimilarity values).

In order to compare it with the physical layout, the MDS output, consisting of 49 points in 2D space, underwent Procrustes transformation, which uses scaling, translation, rotation, and inversion to fit the points to their physical locations (Sereno and Lehky, 2011). Procrustes Distance (PD) quantifies how different the final, transformed, two-dimensional points (corresponding to the 49 location) are from the physical layout. A low PD means that the 2D projection of the RDM corresponds well to the physical distances.

**Figure 2:**
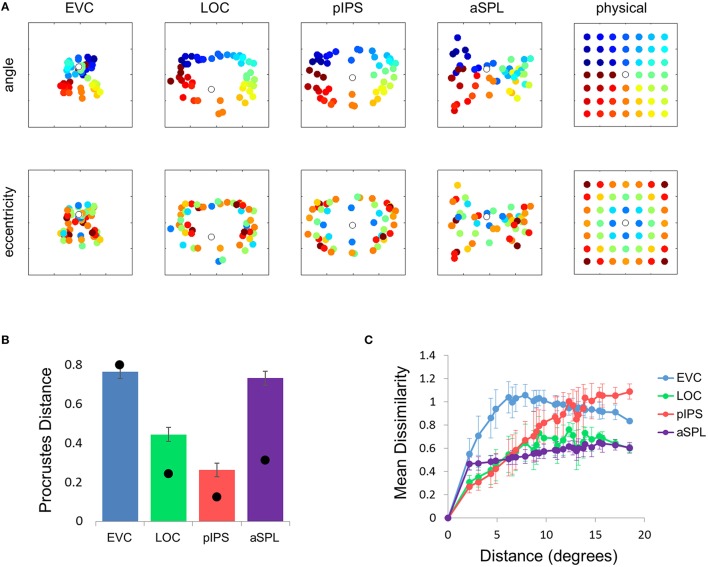
**Multidimensional scaling results. (A)** Multidimensional Scaling (MDS) was performed on the group RDM of each ROI. MDS reduces the dimensionality of the location representation to 2 dimensions such that the distances between the output points correspond as well as possible to the representational dissimilarities. The MDS results were further transformed to fit the physical layout (right-most column) as well as possible, and these results are plotted. Finally, the output points were colored according to the angle (top row) or eccentricity (bottom row) of the corresponding locations. Note that results in pIPS correspond well to the physical layout, while in EVC the MDS results are warped with respect to the true physical layout (e.g., cyan points are between blue and red points). **(B)** Goodness-of-fit of the Procrustes-transformed MDS results to the physical layout was quantified by Procrustes Distance (PD): the better the fit, the smaller the PD. Complementing the results of Figure 1E, MDS results in pIPS fit the physical layout best, and in EVC the fit is the worst. Bars and black points represent single-subject (mean ± SEM) and group results, respectively. **(C)** Group Distance-Dissimilarity (DD) Function. Dissimilarity values of the RDM are plotted as a function of distance, averaging over all pairs of locations at the same distance, ± the standard deviation. Note that for a distance of zero, dissimilarity is by definition 0.

**Figure 3:**
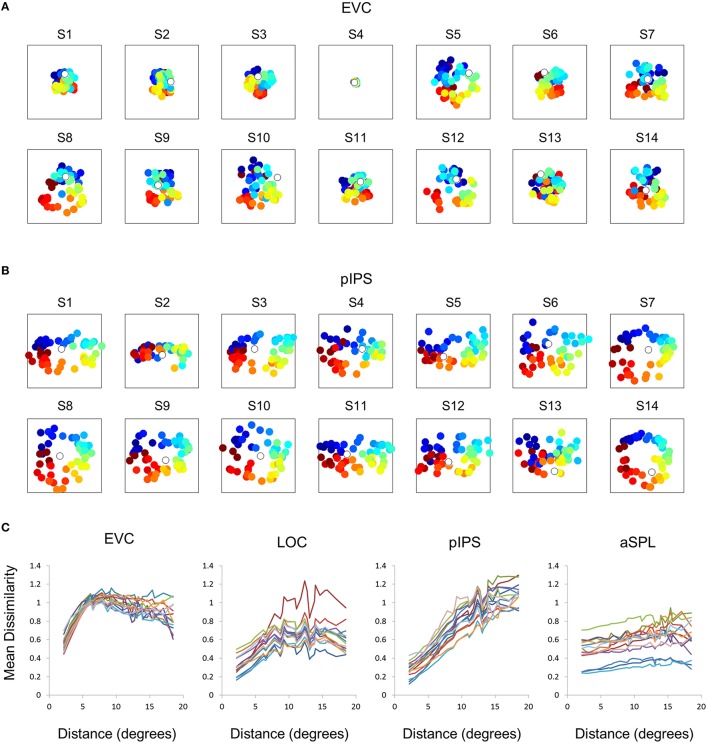
**Single subject MDS results and DD functions. (A)** Single subject MDS results in EVC. Most subjects display the spatial warping apparent in the group RDM (e.g., cyan points are between blue and red points). **(B)** Single subject MDS results in pIPS. For most subjects the representation corresponds well to the physical layout (Figure 2A, top right). **(C)** Single subject DD functions. The function shape is very similar across subjects, for each of the ROIs. Note that for a distance of zero, dissimilarity is by definition 0; We therefore ignore the first data-point in these plots.

This visualization reveals a warping in the spatial representation of EVC (Figure 2A). At first glance it may seem that in all four ROIs the MDS layout is consistent (more or less) with the physical layout, with the aSPL layout noisier than the others, and the EVC layout scaled down. Note, however, that these results are after performing Procrustes transformation which includes scaling—so the scaling presented is optimal, and brings the final layout as close as possible to the physical layout. The fact that the EVC layout is smaller than the others indicates instead that it does not correspond well to the physical layout. Furthermore, on closer inspection of the EVC result it is clear that the resulting layout is warped, or non-topological. For example, while the locations marked by cyan are physically located between the blue and yellow locations, the EVC layout places them between the blue and red locations. This warping is evident both at the group level and for single subject RDMs (Figures 2A, 3A). Spatial representations in pIPS, on the other hand, reflect the physical locations relatively well (Figures 2A, 3B).

The PD results confirmed the impression that in EVC the spatial representation is incongruent with the physical locations and distances: while pIPS displayed the lowest PD, EVC showed the highest PD (Figure 2B).

### Distance-dissimilarity function

The spatial warping in EVC can be seen vividly when plotting dissimilarity as a function of physical distance—the Distance-Dissimilarity (DD) function (Fischer and Whitney, 2009). In pIPS this function is close to monotonic—larger distances between locations correspond to larger dissimilarities between their representations in pIPS. This means that in pIPS the dissimilarity between patterns carries information regarding the distance between the corresponding locations. In EVC, however, this is not the case. For small distances, dissimilarity rises with physical distance. For large distances however, the opposite is true, and dissimilarity actually drops with distance, after reaching a maximum at distances of ~8° (Figure 2C). As with the previous analysis, this phenomenon is evident both at the group level and for single subjects (Figure 3C). Thus, EVC carries accurate discriminative information, enabling one to tell whether two objects are located in the same location or not. However, in the case that the two locations are different, the similarity of representations in EVC does not reflect the true distance between the locations, since locations further away from one another tend to have a higher similarity than locations closer to each other.

### Computational model

**Figure 4:**
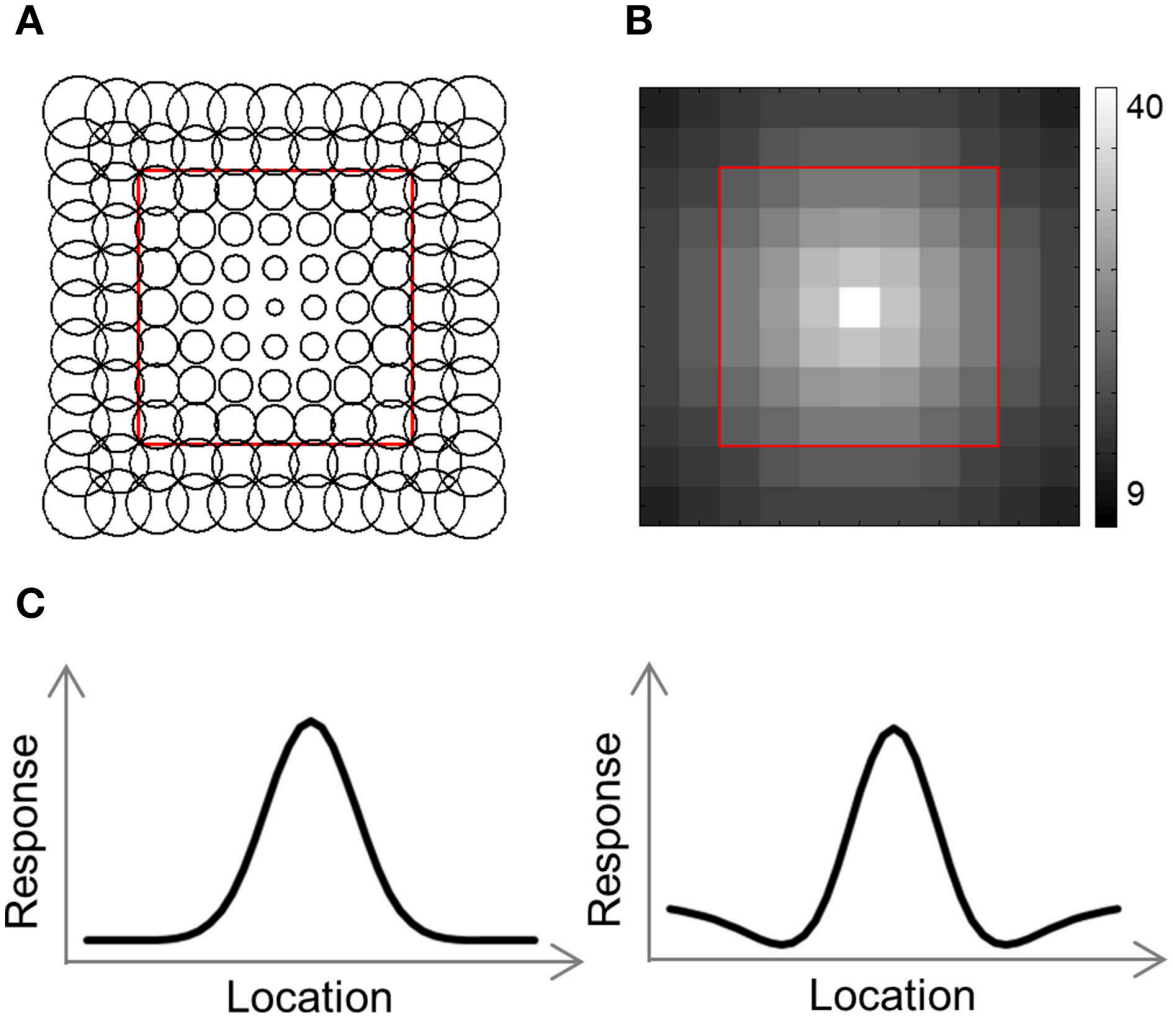
**Computational model outline. (A)** The model consisted of an array of RFs. Adjacent RFs were spaced 1 stimulus width apart. The RF size was fitted to each RDM and increased linearly with eccentricity. **(B)** Number of RFs covering each location. The number of RFs decreased exponentially with eccentricity. **(C)** We used two versions of the model, illustrated here by one-dimensional profiles of the RF shape. In the Gaussian model, each RF was a 2-dimensional Gaussian (left), while in the DoG model each RF included an inhibitory surround region (right).

**Figure 5:**
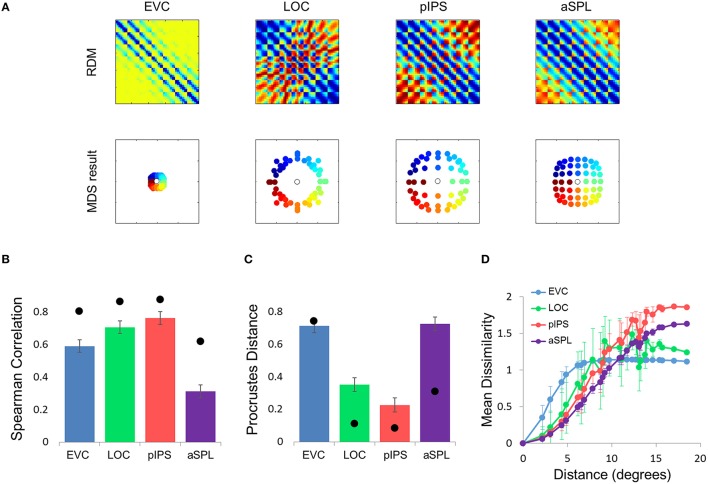
**Gaussian model results. (A)** Model RDMs produced for each ROI (top row) and their matching MDS results (bottom row). Notice that the model does not reproduce the spatial warping evident in the EVC MDS result (compare the bottom left MDS result with Figure 2A, bottom left). **(B)** RDM correlations for the Gaussian model. The correlation reflects the similarity between the ROI RDM and the model RDM. **(C)** PD for the Gaussian model. The PD reflects the difference between the ROI MDS layout and the model MDS layout. In **(B,C)** bars and black points represent single-subject (mean ± SEM) and group results, respectively. **(D)** Gaussian Model Distance-Dissimilarity (DD) Function. Dissimilarity values of the RDM are plotted as a function of distance, averaging over all pairs of locations at the same distance, ± the standard deviation. The dissimilarity values are calculated for the model as for the group data (Figure 2C).

What is the cause for the non-monotonic DD function, warped spatial layout and low levels of relative information in EVC? One obvious suspect is the population receptive field (pRF) size. As it is known that pRF size increases along both the ventral and dorsal streams (Amano et al., 2009; Sprague and Serences, 2013; Kay et al., 2015), it is likely that the ROIs showing higher levels of relative information (i.e., pIPS and LOC) have larger pRFs than EVC. We thus hypothesized that differences in pRF size lie at the root of the differences in relative information found between ROIs (Lehky and Sereno, 2011). To test this hypothesis we built a computational model comprised of an array of RFs spread across the visual field (Figures 4A,B), with two free parameters determining the pRF sizes. We then fitted the model to each RDM. When we fitted the free parameters to each ROI the model succeeded in creating RDMs (Figure 5A) that were similar to the data RDMs, as can be seen in the correlations between data and model RDMs (Figure 5B). Furthermore, the model's DD functions were similar to the data DD functions as well (Figure 5D). Specifically, when fit to the EVC RDM, the model indeed exhibited a non-monotonic DD function, plateauing (and dropping slightly) after reaching a maximum at ~8°, similar to the data (compare to Figure 2C). However, the model was unable to recreate the warping in the spatial representation in EVC: the colors in the MDS result of the model fit to EVC (Figure 5A, left) are in order, meaning that nearby locations have similar representations. This is contrast to the EVC MDS layout (Figure 2A, left), which shows a clear warping—the cyan points are between the blue and red points, even though the corresponding locations are located between the blue and yellow locations.

**Figure 6:**
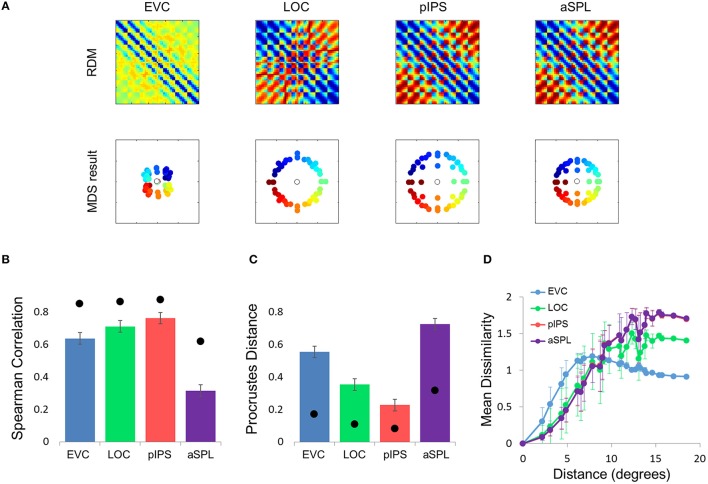
**Difference-of-Gaussians (DoG) model results. (A)** Model RDMs produced for each ROI (top row) and their matching MDS results (bottom row). Notice that, in contrast to the Gaussian model, when fit to the EVC data, the DoG model succeeds in reproducing the spatial warping evident in Figure 2A. **(B)** RDM correlations for the DoG model. The correlation reflects the similarity between the ROI RDM and the model RDM. **(C)** PD for the DoG model. The PD reflects the difference between the ROI MDS layout and the model MDS layout. In **(B,C)** bars and black points represent single-subject (mean ± SEM) and group results, respectively. The PD for the EVC group MDS results are considerably lower here than for the Gaussian model (Figure 5C). **(D)** DD function for the DoG model (the pIPS function is mostly occluded by the aSPL function). Dissimilarity values of the RDM are plotted as a function of distance, averaging over all pairs of locations at the same distance, ± the standard deviation. The dissimilarity values are calculated for the model as for the group data (Figure 2C).

To better model the EVC spatial representation, we extended the model to consist of an array of Difference-of-Gaussian (DoG) RFs (Figure 4C). It has been shown recently that in EVC, DoG provides a better fit to fMRI voxel pRFs (Zuiderbaan et al., 2012). We therefore, hypothesized that a model of DoG RFs would better fit the EVC data. Indeed, the DoG model fit better than the Gaussian model to the EVC data (Figures 6B-D). Furthermore, this model succeeded in reproducing the spatial warping seen in the EVC MDS results (Figure 6A, left).

Taken together, these results demonstrate that the non-monotonic DD function can be modeled by an array of Gaussian RFs. The reason for the non-monotonicity in this case is that large distances between locations usually involve peripheral locations, which evoke responses in fewer RFs than foveal locations. Correlating the activation patterns of peripheral locations results in lower dissimilarities than for locations closer to one another which activate more RFs (consider that if two distant locations do not activate a RF, its response to both locations is identical). This model, however, cannot explain the spatial warping in EVC. To reproduce this warping we had to replace the Gaussian RFs with DoG RFs, which have an inhibitory surround.

### Searchlight analysis

**Figure 7:**
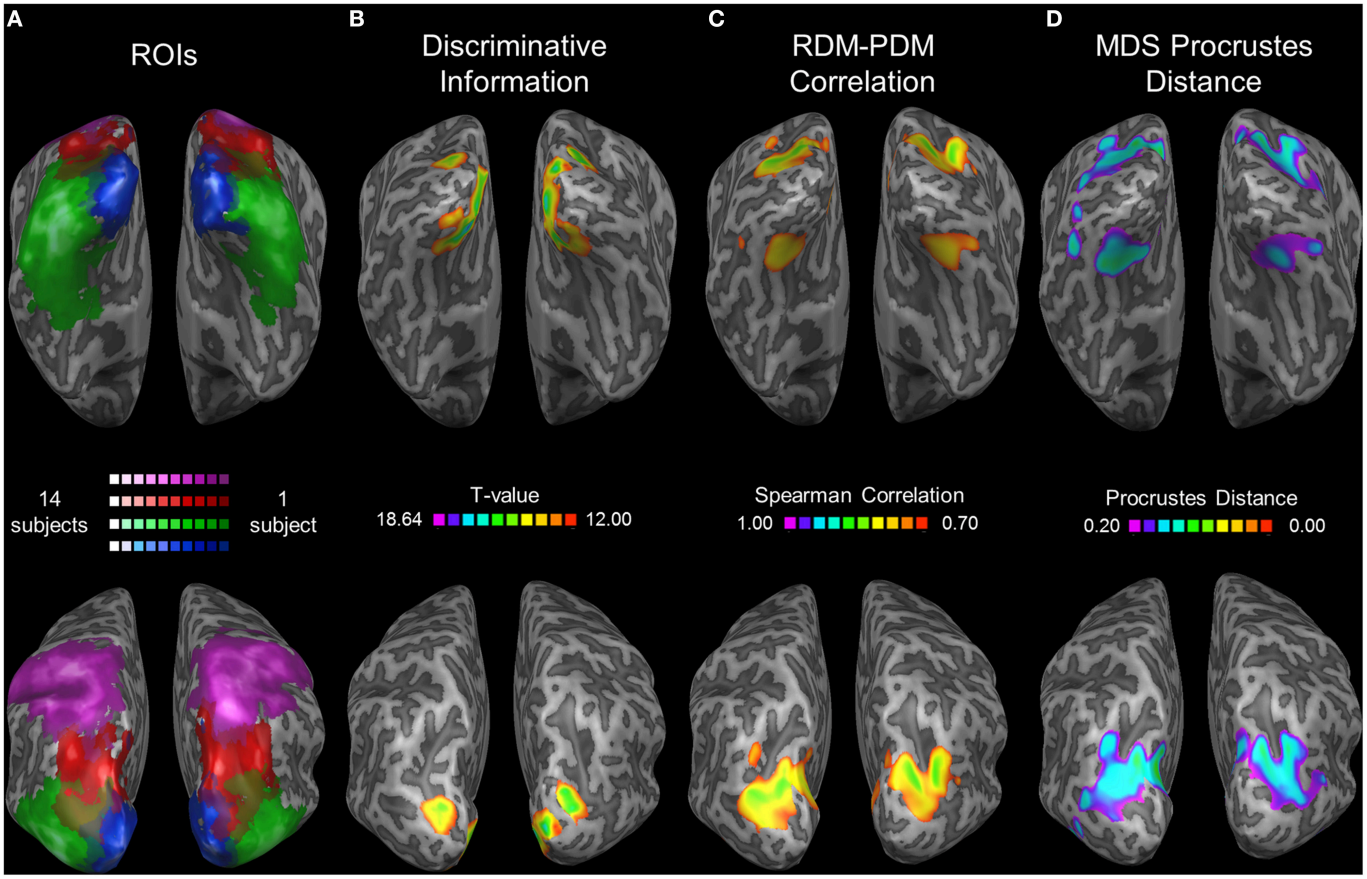
**Searchlight results. (A)** Group ROI probability map of EVC (blue), LOC (green), pIPS (red), and aSPL (purple). All subjects' ROIs were combined and are presented as a single ROI for each region. Brighter colors represent areas (in Talairach coordinates) that are included in a higher number of subjects' ROIs. **(B–D)** Searchlight results (searchlight size: 7 × 7 × 7 voxels). Top: ventral view. Bottom: dorsolateral view. Thresholds were chosen in order to present clusters without many small isolated areas. **(B)** Discriminative Location Information, analyzed by the correlation method (see Roth and Zohary, 2015b for details). At each voxel, an analysis of discriminative information (termed the correlation method) was performed upon each subject's data, and the voxels showing the highest information levels across subjects are presented here (colored according to the *t*-values; Higher *t*-values indicate higher levels of information), overlaid on one subject's inflated cortex. Results for a smaller searchlight (5 × 5 × 5 voxels) are presented in Roth and Zohary (2015b). **(C)** Relative Location Information, measured by Spearman Correlation between the group RDM and the PDM. Higher values indicate better correspondence between the 2 matrices. **(D)** Relative Location Information, measured by Procrustes Distance (PD) between the group MDS result and the physical layout. Note that a *lower* PD represents *more* relative information.

To extend our analysis to regions not included in our ROIs we performed a whole-brain searchlight analysis. We found the highest levels of correlation between the RDM and PDM in a parietal region coinciding with pIPS (see the group ROI probability maps in Figure 7A) and in a ventral region on the medial border of LOC (Figure 7C). Moreover, these same regions exhibit the lowest PD levels for the MDS analysis (Figure 7D). It seems, therefore, that in the dorsal stream we localized the most relevant region, with the highest levels of relative location information. Furthermore, this area showed the highest levels of discriminative information in the dorsal stream as well (Figure 7B). In the ventral stream, however, the relative information searchlight results differ from the discriminative results, and it seems that LOC is not centered on the region showing the highest level of relative information. Instead, voxels showing peak relative information in the ventral stream were located nearby the fusiform face area (Talairach coordinates for the peak informative voxel: RDM-PDM correlation: Right hemisphere [29, −68, −12]; Left [−31, −65, −15]; PD: Right [32, −65, 0]; Left [−28, −65, −3]. Compare with FFA coordinates reported by Kanwisher et al. (1997): [40, −55, −10] and [40, −55, −10] for right and left hemispheres respectively).

### Displacement population vectors

**Figure 8:**
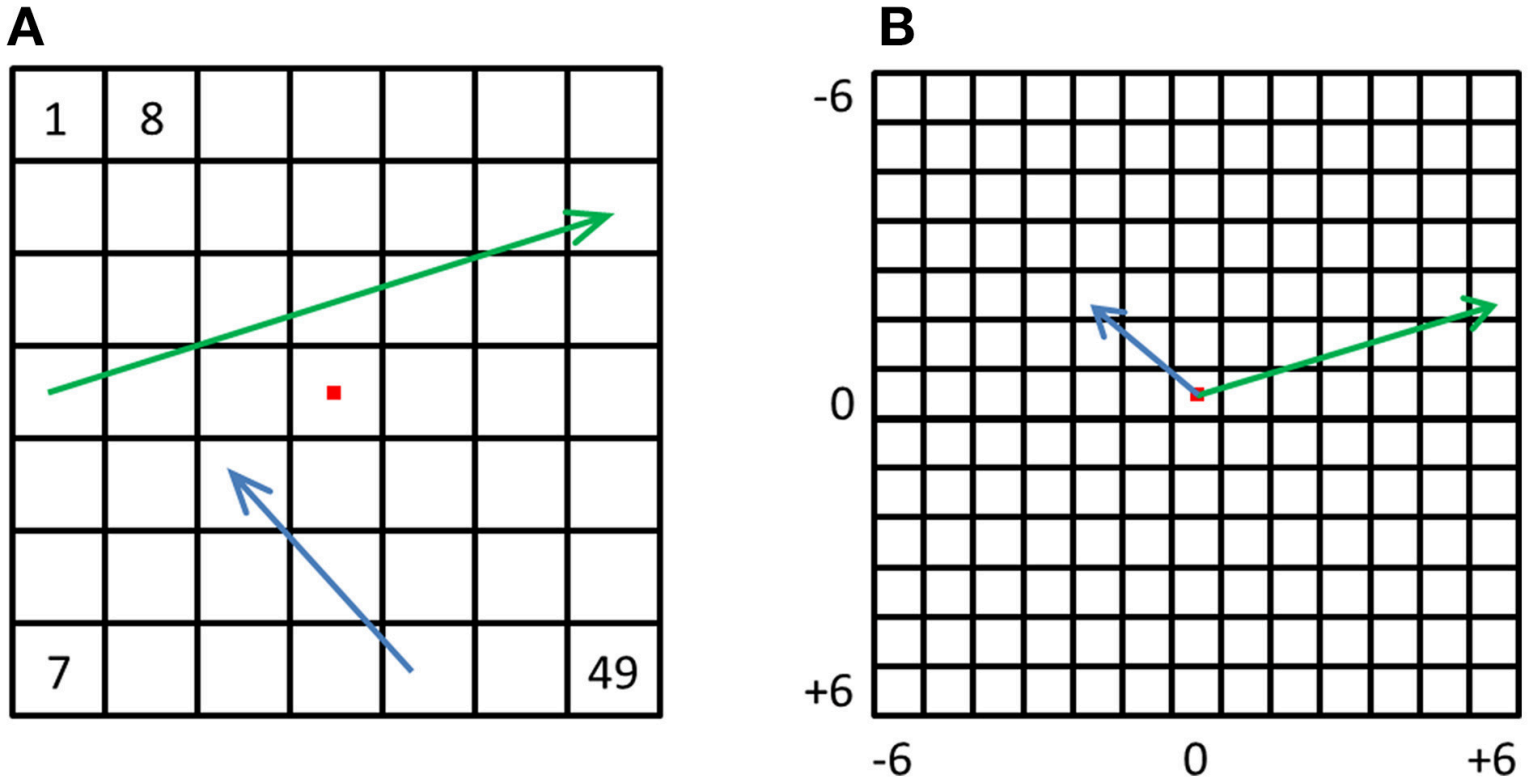
**Displacement vectors. (A)** For every possible displacement we created a synthetic population vector and a corresponding physical vector. The green and blue arrows depict two such physical vectors. **(B)** All physical vectors were aligned to a single origin, and the distances were computed between all pairs, resulting in a dPDM. The dRDM was composed of dissimilarities between all pairs of synthetic vectors.

**Figure 9:**
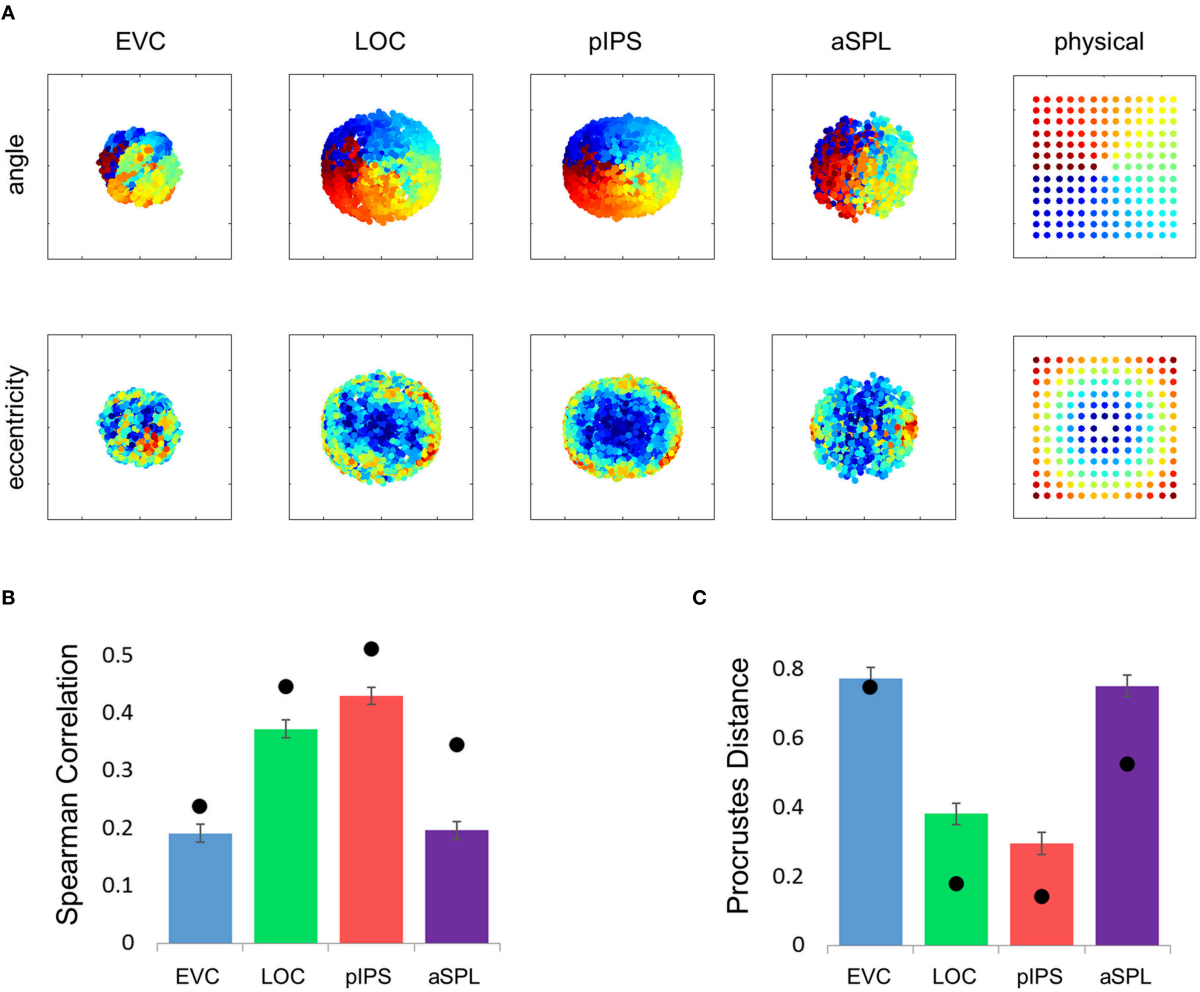
**Displacement representations. (A)** The MDS results transformed to fit the physical layout (right-most column) as well as possible. The output points were colored according to the angle (top row) or eccentricity (bottom row) of the corresponding locations. As with the distance analysis (Figure 2) pIPS shows a good correspondence to the physical layout, while in EVC the MDS results do not fit the physical layout well. **(B)** dRDM-dPDM Spearman correlation results. Results for displacement vectors are similar to the location RDM results in Figure 1E. **(C)** Goodness-of-fit of the Procrustes-transformed dMDS results to the physical layout (dPDM) quantified by PD. Results are similar to the location MDS results in Figure 2B. In **(B,C)** bars and black points represent single-subject (mean ± SEM) and group results, respectively.

Until now we focused on relative location information and showed that pIPS, but not EVC, carries reliable information regarding the distances between locations. However, many times during daily life we need to know not only the distance between objects but also the direction from one object to the other, e.g., during hand-object interactions. Do visual regions carry information regarding the direction from one location to another? To answer this question we created displacement population vectors representing the possible changes in location by subtracting location representations from one another (Figure 8). We then tested how well dissimilarities between these displacement representations reflect the distances between the physical location displacements, by both correlating the resulting dRDM with the dPDM (Figure 9B), and by performing MDS and calculating the PD between the output and the physical layout (Figures 9A,C). Results were similar to the location analyses, showing an accurate representation in pIPS, but not in EVC. We thus conclude that population vectors in pIPS carry information not only regarding the location of a presented stimulus relative to other possible locations, but also regarding the distance and direction from the current (i.e., stimulated) location to other locations.

## Discussion

### Results

Using fMRI, we measured brain activity in 14 individuals while viewing video clips presented at 49 different retinal locations. We studied the patterns of activity in visual regions and measured relative location information. Our results indicate that fMRI activation patterns reflect relative physical locations in pIPS, but not in EVC. In EVC, patterns of nearby locations are less similar than more distant locations, creating a non-monotonic dissimilarity-distance function, whereas in pIPS similarity drops with distance. These results are in contrast to our previous results of discriminative information showing the highest levels of information in EVC (Roth and Zohary, 2015b), suggesting a dissociation between discriminative and relative location information. Finally, we found that these results extend to spatial displacements as well; relative directions, as well as distances, are preserved and represented accurately in pIPS, but are warped in EVC. A simple computational model consisting of an array of RFs suggests that the differences between ROIs derive from differences in pRF size and shape. When combined with our previous findings (Roth and Zohary, 2015b), these results suggest a transformation along the visual hierarchy, from a low-level location-discriminable representation in EVC, to high-level spatially accurate representations in pIPS and LOC, before reaching an almost location-invariant representation in aSPL.

The difference between the spatial representations in EVC and pIPS may reflect the different functional roles of these regions. Posterior IPS is involved in action understanding (Shmuelof and Zohary, 2005; Culham and Valyear, 2006; Lestou et al., 2008), which frequently involves interactions between objects, and for which relative location information (such as the location of a hand relative to a cup handle) is highly relevant. It therefore makes sense that we would find such information in pIPS.

The ventral stream, on the other hand, may make use of relative information in order to enable object recognition. In many cases, object recognition makes use of relative locations, i.e., distances and directions between different object-parts, such as for configural processing of faces where the spacing between features plays a critical role (Maurer et al., 2002). The area we found nearby the FFA in the searchlight analysis may be involved in such processing. EVC might not be directly involved in such processing, and therefore may not take a part in computing relative distances.

### Limitations

One major concern relevant to all MVPA studies is the degree to which the voxel population vectors reflect neural population vectors (Kriegeskorte et al., 2008b; Dubois et al., 2015).

With regard to location information, although fMRI patterns in EVC show low levels of relative location information, electrophysiologically measured patterns of neuronal spiking activity might show higher levels of information in EVC. This could occur, for example, if relative location information is carried in EVC output (i.e., spiking) rather than in EVC synaptic activity, which is suggested to be the source of the BOLD signal (Logothetis et al., 2001). Nevertheless, despite significant differences between fMRI measurements and neural recordings, we believe the two methods should reach similar results with regard to location information (at least in EVC). The most robust coarse-scale organization principle throughout visual cortex is an orderly mapping of stimulus location in retinotopic coordinates (Wandell et al., 2007). As a result, the variability of receptive field centers in each voxel is relatively small. Therefore, the receptive field of each neuron in a voxel should be relatively similar to the voxel population receptive field, and the voxel patterns of activity should correspond closely to the neural patterns of activity. This claim is supported by the close correspondence between neural receptive field sizes and voxel population receptive field (pRF) sizes across visual cortex (Dumoulin and Wandell, 2008).

An additional limitation of our study relates to the eccentricities used. The most peripheral stimuli used in this study were centered 9.2° from fixation, and the largest distance between locations was 18.4°. We therefore, do not know what the responses would be to more peripheral stimuli, or across wider distances. Specifically, we cannot conclude that the DD function in pIPS remains monotonic for larger eccentricities; the function may reach a peak at around 18° and then start dropping, similar to what happens in EVC (which peaks at ~8°).

A further limitation is that we stimulated locations sequentially and not simultaneously. Arguably, in order to study representations of distances and directions between locations it is necessary to stimulate both locations at the same time. However, stimulating two locations simultaneously would introduce several problematic factors into the experiment. First, it is unclear whether attention can be directed at two locations simultaneously (Jans et al., 2010; Landau and Fries, 2012). Therefore, subjects would probably pay more attention to one of the locations, and the activity pattern would reflect that location more than the second simultaneously stimulated location. Furthermore, in such an experiment it would be impractical to stimulate all possible pairs of locations, using our 7 × 7 location grid, since there are 2352 possible pairs. Finally, it is still unknown how multiple simultaneous stimuli interact at the voxel level. Assuming a voxel has a response *r*_1_ to location *s*_1_ and a response *r*_2_ to location *s*_2_, the response evoked by simultaneous stimulation of locations *s*_1_ and *s*_1_ might be a linear summation of the two responses *r*_1_ + *r*_2_ (MacEvoy and Epstein, 2009). However, the evoked response may instead be a sublinear or supralinear summation of the responses to the individual locations, *a*(*r*_1_ + *r*_2_), where *a* < 1 or *a* > 1, respectively (Baeck et al., 2013; Orhan and Ma, 2015). Since the type of summation for each voxel is unknown, it would be difficult to determine what part of the observed response was evoked by which location. Future studies aimed at studying relative location information by stimulating multiple locations simultaneously will have to overcome these difficulties.

### Correspondence to psychophysical findings

There exists psychophysical evidence that supports a dissociation between relative and discriminative location information. Mateeff and Gourevich (1983) conducted a series of psychophysical experiments in order to study behavioral systematic mislocalization. In one experiment (Experiment 1) subjects fixated centrally and were presented with a brief flash of light at a variable eccentricity. Aided by a fixed scale, subjects reported the perceived stimulus location. In this experiment, consistent with other studies (Hagenaar and van der Heijden, 1997; Müsseler et al., 1999; Eggert et al., 2001), subjects reported that the light spot was closer to fixation than it actually was, and this error grew with eccentricity. In another experiment (Experiment 5) subjects fixated on a central light spot, while another light spot was present at a fixed distance to the left of fixation. A third light spot was flashed briefly, at a variable distance to the left of the second point, and subjects had to judge whether the distance between fixation and the second spot was larger than that from the second to the third point. In this experiment no systematic bias was evident, even though the comparison was between two distances at different eccentricities. Combined results of the experiments indicate that comparison of distances does not necessarily rely on location estimation. Indeed, the authors suggest that the two tasks may involve independent processes, namely, perception of location and perception of distance. These processes rely on the two types of information we studied; Location perception depends on determining which number on the scale is at the same location as the light flash (i.e., discriminative information), while distance perception relies upon the comparison of two distances between objects (i.e., relative information). Thus, our results agree with the authors' suggestion, and provide further evidence of a dissociation between these two forms of information.

### Comparison to electrophysiology in monkeys

In a recent electrophysiological study conducted on macaque monkeys, Sereno and Lehky measured neural responses to eight locations at fixed angles, and found, using an MDS analysis similar to that described above, that dorsal stream representations in lateral intraparietal cortex (LIP) match physical distances better than ventral stream representations in anterior inferotemporal cortex (AIT) (Lehky and Sereno, 2007; Sereno and Lehky, 2011). Although major methodological differences exist between the studies (see Section Limitations, above), these results correspond closely with our ROI analysis which found the highest levels of relative location information in the dorsal stream, specifically in the intraparietal sulcus. Our searchlight results further suggest that an additional region in the ventral stream may have levels of relative information on par with the dorsal stream.

### Conclusions and further directions

We analyzed the amount of relative location information present in fMRI activation patterns. We found that while EVC carries the highest levels of discriminative information, relative information levels in EVC are low relative to higher-order areas, and the spatial representations there are warped. Using a computational model we showed that pRF size influences the amount of relative information and that the spatial warping in EVC may be a result of DoG pRFs which include inhibitory surround regions. Our results suggest that representations of the visual field are transformed along both visual streams, and warrant future investigation of discriminative and relative information with regard to other visual features.

The dissociation we found between the two types of location information raises the question: do similar dissociations, between discriminative and relative information, exist with respect to other stimulus properties? Regarding color, Brouwer and Heeger (2009) found a similar dissociation in V1. Patterns of responses in V1 carried information that discriminated between different colors, but principal component analysis revealed that similarity between responses did not reflect the physical similarity between colors. This was in contrast to V4 where patterns of activity both discriminated between colors and enabled an accurate representation of the color space. Future studies may make use of existing datasets to analyze relative information with regard to additional visual features, including low-level features such as orientation (Kamitani and Tong, 2005) or motion (Kamitani and Tong, 2006), and higher-level features such as shape (Drucker and Aguirre, 2009) or geographical location (Morgan et al., 2011).

## Author contributions

The author designed the experiment, collected and analyzed the data, wrote the manuscript, and approved it for publication.

### Conflict of interest statement

The author declares that the research was conducted in the absence of any commercial or financial relationships that could be construed as a potential conflict of interest.

## References

[B1] Amano K. Wandell B. A. Dumoulin S. O. (2009). Visual field maps, population receptive field sizes, and visual field coverage in the human MT+ complex. J. Neurophysiol. 102, 2704–2718. 10.1152/jn.00102.2009 19587323 PMC2777836

[B2] Andersen R. A. Essick G. K. Siegel R. M. (1985). Encoding of spatial location by posterior parietal neurons. Science 230, 456–458. 10.1126/science.4048942 4048942

[B3] Andersen R. A. Snyder L. H. Bradley D. C. Xing J. (1997). Multimodal representation of space in the posterior parietal cortex and its use in planning movements. Annu. Rev. Neurosci. 20, 303–330. 10.1146/annurev.neuro.20.1.303 9056716

[B4] Baeck A. Wagemans J. de Beeck H. P. O. (2013). The distributed representation of random and meaningful object pairs in human occipitotemporal cortex: the weighted average as a general rule. Neuroimage 70, 37–47. 10.1016/j.neuroimage.2012.12.023 23266747

[B5] Brouwer G. J. Heeger D. J. (2009). Decoding and reconstructing color from responses in human visual cortex. J. Neurosci. 29, 13992–14003. 10.1523/JNEUROSCI.3577-09.2009 19890009 PMC2799419

[B6] Cichy R. M. Sterzer P. Heinzle J. Elliott L. T. Ramirez F. Haynes J. D. (2013). Probing principles of large-scale object representation: category preference and location encoding. Hum. Brain Mapp. 34, 1636–1651. 10.1002/hbm.22020 22371355 PMC6870376

[B7] Cohen Y. E. Andersen R. A. (2002). A common reference frame for movement plans in the posterior parietal cortex. Nat. Rev. Neurosci. 3, 553–562. 10.1038/nrn873 12094211

[B8] Colby C. L. Goldberg M. E. (1999). Space and attention in parietal cortex. Annu. Rev. Neurosci. 22, 319–349. 10.1146/annurev.neuro.22.1.319 10202542

[B9] Culham J. C. Valyear K. F. (2006). Human parietal cortex in action. Curr. Opin. Neurobiol. 16, 205–212. 10.1016/j.conb.2006.03.005 16563735

[B10] Drucker D. M. Aguirre G. K. (2009). Different spatial scales of shape similarity representation in lateral and ventral LOC. Cereb. Cortex 19, 2269–2280. 10.1093/cercor/bhn244 19176637 PMC2742590

[B11] Dubois J. de Berker A. O. Tsao D. Y. (2015). Single-unit recordings in the macaque face patch system reveal limitations of fMRI MVPA. J. Neurosci. 35, 2791–2802. 10.1523/JNEUROSCI.4037-14.2015 25673866 PMC4323541

[B12] Dumoulin S. O. Wandell B. A. (2008). Population receptive field estimates in human visual cortex. Neuroimage 39, 647–660. 10.1016/j.neuroimage.2007.09.034 17977024 PMC3073038

[B13] Eggert T. Ditterich J. Straube A. (2001). Mislocalization of peripheral targets during fixation. Vision Res. 41, 343–352. 10.1016/S0042-6989(00)00263-7 11164449

[B14] Fischer J. Whitney D. (2009). Attention narrows position tuning of population responses in V1. Curr. Biol. 19, 1356–1361. 10.1016/j.cub.2009.06.059 19631540 PMC2757109

[B15] Golomb J. D. Kanwisher N. (2012). Higher level visual cortex represents retinotopic, not spatiotopic, object location. Cereb. Cortex 22, 2794–2810. 10.1093/cercor/bhr357 22190434 PMC3491766

[B16] Golomb J. D. Kupitz C. N. Thiemann C. T. (2014). The influence of object location on identity: a “spatial congruency bias.” J. Exp. Psychol. 143, 2262. 10.1037/xge0000017 25222263

[B17] Hagenaar R. van der Heijden A. (1997). Location errors in partial-report bar-probe experiments: in search of the origin of cue-alignment problems. Mem. Cognit. 25, 641–652. 10.3758/BF03211305 9337582

[B18] Haxby J. V. Gobbini M. I. Furey M. L. Ishai A. Schouten J. L. Pietrini P. (2001). Distributed and overlapping representations of faces and objects in ventral temporal cortex. Science 293, 2425–2430. 10.1126/science.1063736 11577229

[B19] Hayworth K. J. Lescroart M. D. Biederman I. (2011). Neural encoding of relative position. J. Exp. Psychol. Hum. Percept. Perform. 37, 1032. 10.1037/a0022338 21517211

[B20] Jans B. Peters J. C. De Weerd P. (2010). Visual spatial attention to multiple locations at once: the jury is still out. Psychol. Rev. 117, 637. 10.1037/a0019082 20438241

[B21] Kamitani Y. Tong F. (2005). Decoding the visual and subjective contents of the human brain. Nat. Neurosci. 8, 679–685. 10.1038/nn1444 15852014 PMC1808230

[B22] Kamitani Y. Tong F. (2006). Decoding seen and attended motion directions from activity in the human visual cortex. Curr. Biol. 16, 1096–1102. 10.1016/j.cub.2006.04.003 16753563 PMC1635016

[B23] Kanwisher N. McDermott J. Chun M. M. (1997). The fusiform face area: a module in human extrastriate cortex specialized for face perception. J. Neurosci. 17, 4302–4311. 9151747 10.1523/JNEUROSCI.17-11-04302.1997 PMC6573547

[B24] Kay K. N. Weiner K. S. Grill-Spector K. (2015). Attention reduces spatial uncertainty in human ventral temporal cortex. Curr. Biol. 25, 595–600. 10.1016/j.cub.2014.12.050 25702580 PMC4348205

[B25] Kravitz D. J. Kriegeskorte N. Baker C. I. (2010). High-level visual object representations are constrained by position. Cereb. Cortex 20, 2916–2925. 10.1093/cercor/bhq042 20351021 PMC2978243

[B26] Kriegeskorte N. Goebel R. Bandettini P. (2006). Information-based functional brain mapping. Proc. Natl. Acad. Sci. U.S.A. 103, 3863–3868. 10.1073/pnas.0600244103 16537458 PMC1383651

[B27] Kriegeskorte N. Kievit R. A. (2013). Representational geometry: integrating cognition, computation, and the brain. Trends Cogn. Sci. 17, 401–412. 10.1016/j.tics.2013.06.007 23876494 PMC3730178

[B28] Kriegeskorte N. Mur M. Bandettini P. (2008a). Representational similarity analysis–connecting the branches of systems neuroscience. Front. Syst. Neurosci. 2:4. 10.3389/neuro.06.004.2008 19104670 PMC2605405

[B29] Kriegeskorte N. Mur M. Ruff D. A. Kiani R. Bodurka J. Esteky H.. (2008b). Matching categorical object representations in inferior temporal cortex of man and monkey. Neuron 60, 1126–1141. 10.1016/j.neuron.2008.10.043 19109916 PMC3143574

[B30] Landau A. N. Fries P. (2012). Attention samples stimuli rhythmically. Curr. Biol. 22, 1000–1004. 10.1016/j.cub.2012.03.054 22633805

[B31] Lehky S. R. Sereno A. B. (2007). Comparison of shape encoding in primate dorsal and ventral visual pathways. J. Neurophysiol. 97, 307–319. 10.1152/jn.00168.2006 17021033

[B32] Lehky S. R. Sereno A. B. (2011). Population coding of visual space: modeling. Front. Comput. Neurosci. 4:155. 10.3389/fncom.2010.00155 21344012 PMC3034232

[B33] Lestou V. Pollick F. E. Kourtzi Z. (2008). Neural substrates for action understanding at different description levels in the human brain. J. Cogn. Neurosci. 20, 324–341. 10.1162/jocn.2008.20021 18275338

[B34] Logothetis N. K. Pauls J. Augath M. Trinath T. Oeltermann A. (2001). Neurophysiological investigation of the basis of the fMRI signal. Nature 412, 150–157. 10.1038/35084005 11449264

[B35] MacEvoy S. P. Epstein R. A. (2009). Decoding the representation of multiple simultaneous objects in human occipitotemporal cortex. Curr. Biol. 19, 943–947. 10.1016/j.cub.2009.04.020 19446454 PMC2875119

[B36] Marre O. Botella-Soler V. Simmons K. D. Mora T. Tkačik G. Berry M. J. II. (2014). High accuracy decoding of dynamical motion from a large retinal population. PLoS Comput. Biol. 11:e1004304. 10.1371/journal.pcbi.1004304 26132103 PMC4489022

[B37] Mateeff S. Gourevich A. (1983). Peripheral vision and perceived visual direction. Biol. Cybern. 49, 111–118. 10.1007/BF00320391 6661443

[B38] Maurer D. Le Grand R. Mondloch C. J. (2002). The many faces of configural processing. Trends Cogn. Sci. 6, 255–260. 10.1016/S1364-6613(02)01903-4 12039607

[B39] Morgan L. K. MacEvoy S. P. Aguirre G. K. Epstein R. A. (2011). Distances between real-world locations are represented in the human hippocampus. J. Neurosci. 31, 1238–1245. 10.1523/JNEUROSCI.4667-10.2011 21273408 PMC3074276

[B40] Müsseler J. van der Heijden A. H. Mahmud S. Deubel H. Ertsey S. (1999). Relative mislocalization of briefly presented stimuli in the retinal periphery. Percept. Psychophys. 61, 1646–1661. 10.3758/BF03213124 10598476

[B41] Orhan A. E. Ma W. J. (2015). Neural population coding of multiple stimuli. J. Neurosci. 35, 3825–3841. 10.1523/JNEUROSCI.4097-14.2015 25740513 PMC4461696

[B42] Pertzov Y. Husain M. (2014). The privileged role of location in visual working memory. Atten. Percept. Psychophys. 76, 1914–1924. 10.3758/s13414-013-0541-y 24027033 PMC4212176

[B43] Roth Z. N. Zohary E. (2015a). Fingerprints of learned object recognition seen in the fMRI activation patterns of lateral occipital complex. Cereb. Cortex 25, 2427–2439. 10.1093/cercor/bhu042 24692511

[B44] Roth Z. N. Zohary E. (2015b). Position and identity information available in fMRI patterns of activity in human visual cortex. J. Neurosci. 35, 11559–11571. 10.1523/JNEUROSCI.0752-15.2015 26290233 PMC6605241

[B45] Schwartz G. Macke J. Amodei D. Tang H. Berry M. J. (2012). Low error discrimination using a correlated population code. J. Neurophysiol. 108, 1069–1088. 10.1152/jn.00564.2011 22539825 PMC3424080

[B46] Sereno A. B. Lehky S. R. (2011). Population coding of visual space: comparison of spatial representations in dorsal and ventral pathways. Front. Comput. Neurosci. 4:159. 10.3389/fncom.2010.00159 21344010 PMC3034230

[B47] Shepard R. N. (1980). Multidimensional scaling, tree-fitting, and clustering. Science 210, 390–398. 10.1126/science.210.4468.390 17837406

[B48] Shmuelof L. Zohary E. (2005). Dissociation between ventral and dorsal fMRI activation during object and action recognition. Neuron 47, 457–470. 10.1016/j.neuron.2005.06.034 16055068

[B49] Sprague T. C. Serences J. T. (2013). Attention modulates spatial priority maps in the human occipital, parietal and frontal cortices. Nat. Neurosci. 16, 1879–1887. 10.1038/nn.3574 24212672 PMC3977704

[B50] Treisman A. Zhang W. (2006). Location and binding in visual working memory. Mem. Cognit. 34, 1704–1719. 10.3758/BF03195932 17489296 PMC1868390

[B51] Treisman A. M. Gelade G. (1980). A feature-integration theory of attention. Cogn. Psychol. 12, 97–136. 10.1016/0010-0285(80)90005-5 7351125

[B52] Wandell B. A. Dumoulin S. O. Brewer A. A. (2007). Visual field maps in human cortex. Neuron 56, 366–383. 10.1016/j.neuron.2007.10.012 17964252

[B53] Zuiderbaan W. Harvey B. M. Dumoulin S. O. (2012). Modeling center–surround configurations in population receptive fields using fMRI. J. Vis. 12, 10. 10.1167/12.3.10 22408041

